# Berberine exerts a protective effect on rats with polycystic ovary syndrome by inhibiting the inflammatory response and cell apoptosis

**DOI:** 10.1186/s12958-020-00684-y

**Published:** 2021-01-07

**Authors:** Hao-Ran Shen, Xiao Xu, Xue-Lian Li

**Affiliations:** 1 Department of Gynecology, Obstetrics & Gynecology Hospital of Fudan University, No. 419 Fangxie Road, Shanghai, 200011 P.R. China; 2 Shanghai Key Laboratory of Female Reproductive Endocrine-Related Diseases, No. 419 Fangxie Road, Shanghai, 200011 P.R. China

**Keywords:** Polycystic ovary syndrome, Berberine, Insulin resistance, Testosterone, Cell apoptosis

## Abstract

**Background:**

Polycystic ovary syndrome (PCOS) is a common endocrine disease of the female reproductive system that seriously affects women’s health. Berberine (BBR) has many pharmacological properties and is used as an insulin sensitizer. This study aimed to investigate the effect of BBR on PCOS and explore its related mechanisms.

**Methods:**

Forty-two rats were randomly divided into the following six groups (*n* = 7 per group): control, control + BBR, PCOS-normal diet (ND), PCOS-ND + BBR, PCOS-high-fat diet (HFD), and PCOS-HFD + BBR. The PCOS rat models were established by injecting rats with dehydroepiandrosterone. Further, the rats were gavaged with BBR (150 mg/kg/d) for 6 weeks. Then, the body weight, HOMA-IR, and testosterone levels of all rats were determined. Cell apoptosis of ovary granulosa cells was determined by a TUNEL assay kit. Real-time quantification PCR (RT-qPCR) and western blotting were utilized to evaluate the expression of *TLR4*, *LYN*, *PI3K*, *Akt*, *NF-kB*, *TNF-α*, *IL-1*, *IL-6*, and *caspase-3*.

**Results:**

BBR reduced the levels of insulin resistance and testosterone in PCOS rats. Additionally, the cell apoptosis rate increased significantly in PCOS rats (*P* < 0.05) and decreased after BBR treatment (*P* < 0.05). The results of RT-qPCR and western blotting showed that the expression levels of *TLR4*, *LYN*, *PI3K*, *Akt*, *NF-kB*, *TNF-α*, *IL-1*, *IL-6*, and *caspase-3* significantly increased in PCOS rats, while BBR suppressed their expression levels.

**Conclusions:**

BBR may relieve PCOS pathology and IR values by inhibiting cell apoptosis and by regulating the expression levels of *TLR4*, *LYN*, *PI3K*, *Akt*, *NF-kB*, *TNF-α*, *IL-1*, *IL-6*, and *caspase-3*.

## Background

Polycystic ovary syndrome (PCOS) is one of the most common endocrine disorders, affecting 4–10% of the women of reproductive age [1, 2]. The clinical symptoms of PCOS include irregular menstrual cycles, infertility, hirsutism, acne, insulin resistance (IR), and hyperandrogenemia [3]. PCOS is associated with obesity, diabetes, hypertension, endometrial cancer, and cardiovascular disease [4–7]. Some adverse symptoms of PCOS can be ameliorated by regulating the menstrual cycle (oral contraceptive), by reducing blood testosterone levels (glucocorticoids), by improving IR (metformin), and by inducing ovulation (clomiphene) [8, 9]. However, long-term drug treatments may result in gastrointestinal discomfort, diarrhea, and other serious side effects that adversely affect the patient’s standard of living. Therefore, the development of alternative treatment strategies for women with PCOS is urgently needed.

In recent years, increasing evidence has shown that natural plant-based products may play a role in the treatment and management of PCOS [10, 11]. Berberine (BBR), a small molecule alkaloid isolated from plants, is the major active component of the Chinese herb rhizoma coptidis (Huanglian) [12]. BBR has a variety of pharmacological properties, including anti-inflammatory, antimicrobial, glucose- and cholesterol-reducing, anti-cancer, and immunomodulatory activities [13–15]. A study by Zhang et al. showed that BBR prevented obesity and IR in high-fat diet (HFD)-fed rats by partially modulating the gut microbiota composition [16]. Additionally, another study indicated that the intake of BBR in the short term improved some metabolic and hormonal disorders in women with PCOS, and its related effects might be associated with the changes in body composition in obesity and dyslipidemia [17]. However, the mechanisms of BBR in women with PCOS are currently unclear.

Therefore, in this study, a PCOS rat model was established by injecting rats with dehydroepiandrosterone (DHEA), and the therapeutic effect of BBR on IR in the PCOS model was investigated. Additionally, the potential mechanism of BBR on PCOS was explored by real-time quantification PCR (RT-qPCR) and western blotting. These results will improve our understanding of the development of PCOS, and may provide a novel clinical treatment strategy for PCOS.

## Methods

### Ethics statement

All animal experiments were conducted in strict accordance with the animal ethics standards and with ethics approval from the Ethics Committee of Obstetrics and Gynecology Hospital of Fudan University (no. [2015]45).

### Animals and treatments

Female clean Sprague Dawley rats (6 weeks old, total *n* = 42) were obtained from the Shanghai Laboratory Animal Centre (SLAC, Shanghai, China), and were adaptively fed for 3 days. The PCOS rat models were established following the methods reported by Abramovich et al. [18] with some modifications. The rats were first randomly divided into the following six groups (*n* = 7 per group): control, control + BBR, PCOS-high fat diet (HFD), PCOS-HFD + BBR, PCOS-normal diet (ND), and PCOS-ND + BBR. The rats in the PCOS-HFD and PCOS-HFD + BBR groups were fed with an HFD and were injected with DHEA (source leaf organisms, S24516; 6 mg/100 g body weight with 0.2 ml of sesame oil) for 21 days to induce PCOS in IR rat models. The rats in the PCOS-ND and PCOS-ND + BBR groups were fed with an ND and injected with DHEA. The other rats were fed with an ND and injected with 0.2 ml of sesame oil (control and control + BBR groups). The main energy materials of an ND and an HFD are shown in Table 1. Methylene blue staining was used to examine the changes of vaginal epithelial cells in the DHEA-treated rats under an optical microscope. The PCOS model was considered successful if the vaginal epithelial cells were continuously keratinized for 10 consecutive days [19]. After 12 h of fasting (20,00 to 8:00), the venous blood was extracted from the tails of the PCOS rats. Fasting plasma glucose (FPG) levels were determined by using the ACCU-CHEK Performa glucose [20] meter (Roche Diabetes Care, USA), and the fasting insulin (FINS) levels were determined using rat insulin ELISA kits (Thermo Scientific, USA). In accordance with the homeostasis model assessment of IR (HOMA-IR) method [21], HOMA-IR = FINS × FPG/22.5. HOMA-IR > 2.8 of the PCOS rats was considered to indicate the presence of IR [11].

**Table 1:** The main energy materials of normal diet and high-fat diet (kcal/100 g)

Energy materials	Normal diet	High-fat diet
Protein	90.46	87.20
Carbohydrate	212.96	207.10
Fat	48.58	166.20
In total	352.00	460.50

Subsequently, the rats in the control + BBR, PCOS-HFD + BBR, and PCOS-ND + BBR groups were gavaged with BBR (150 mg/kg/d, Sigma-Aldrich, Louis, MO, USA). The BBR was dissolved with 0.5% sodium carboxymethyl cellulose [Sangon Biotech (Shanghai) Co., LTD, Shanghai, China]. The rats in the other groups were gavaged daily with an equal volume of 0.5% sodium carboxymethyl cellulose. The treatments were administered for 6 weeks.

### Sample collection

At the end of the experiment, the body weights and HOMA-IR of all rats were determined. Further, all rats were euthanized by cervical dislocation, and serum and ovary tissue samples were collected from all rats. Additionally, a part of each ovary tissue sample was fixed in polyformic acid solution. The remaining ovary tissue was stored at − 80 °C for subsequent experiments.

### Histological analysis

For histological analysis, the fixed ovary tissue was embedded in paraffin, sectioned to a thickness of 5 mm, and stained with hematoxylin and eosin (HE). Slides were scanned using the OPTIKA microscope (M.A.D. Co. LTD, Italy) and the morphological changes of the ovary tissues were observed.

### Serum parameter assay

According to the manufacturer’s instructions, the levels of insulin and testosterone in sera were respectively measured using rat insulin and rat testosterone ELISA kits (KGE010, R&D systems, USA).

### Cell apoptosis analysis

The cell apoptosis rate was investigated by using the TUNEL assay kit (Roche Applied Science, Penzberg, Germany). Briefly, slides with 5-μm sections of the ovary tissues were dewaxed by xylene and washed with ethyl alcohol. After sealing with H_2_O_2_ for 30 min, 20 mg/L of protease K was added to the samples. After 20 min, the TUNEL reaction solution was added and incubated for 60 min at 37 °C. Then, anti-luciferin antibodies linked by peroxidase were added and incubated for 30 min at 37 °C. Subsequently, the slides were stained with DAB and restained with hematoxylin. The number of apoptotic cells was observed and calculated under an optical microscope (M.A.D. Co. LTD, Italy). The positive apoptotic cell nuclei were dark brown in color, and the non-apoptotic cell nuclei were blue in color. The Image pro plus 6.0 software was utilized to determine the number of TUNEL-positive cells.

### RT-qPCR analysis

Total RNA in the ovary tissues was isolated using the TRIzol reagent (TIANGEN, Shanghai, China) according to the manufacturer’s instructions. The RNA quantity and quality were evaluated with a microplate reader (Tecan Group LTD, Männedorf, Switzerland). Afterward, cDNA was synthesized using a cDNA Reverse Transcription Kit (TaKaRa Bio Inc., Shiga, Japan) following the manufacturer’s instructions. RT-qPCR was performed using a fluorescence ration PCR instrument (Applied Biosystems Inc., Foster City, CA, USA) with SYBR Green Ι (Thermo, Waltham, MA, USA). The sequences of all primers are shown in Table 2. The PCR conditions were as follows: 3 min at 50 °C, 3 min at 95 °C, 40 cycles of 10 s at 95 °C, 30 s at 60 °C, and 15 s at 72 °C. GAPDH was used as the reference gene.

**Table 2:** The sequences of all primers

Primer	Sequence(5′-3′)
TLR4-rF	GTTCCTTTCCTGCCTGAGACC
TLR4-rR	AGGGTTTCCTGTCAGTACCA
LYN-rF	TGAAGACTCAACCAGTTCCTGA
LYN-rR	TTAGCTTTCCACCACTCCCC
PI3K-rF	GCAAAAGCTTGAAAGCCTGC
PI3K-rR	CAGGGGCTTCTTCTTGGAGG
Akt-rF	TAGCCATTGTGAAGGAGGGC
Akt-rR	CCTGAGGCCGTTCCTTGTAG
NF-κB-rF	TGCCGAGTAAACCGGAACTC
NF-κB-rR	CAGCCAGGTCCCGTGAAATA
TNF-α-rF	ATGGGCTCCCTCTCATCAGT
TNF-α-rR	AAATGGCAAATCGGCTGACG
IL-1-rF	ACAAAAATGCCTCGTGCTGTC
IL-1-rR	GTGCCGTCTTTCATCACACAG
IL-6-rF	TCTGGTCTTCTGGAGTTCCG
IL-6-rR	AGCATTGGAAGTTGGGGTAGG
caspase-3-rF	TACTCTACCGCACCCGGTTA
caspase-3-rR	CGCGTACAGTTTCAGCATGG
CD14-rF	ACTTCTCAGATCCGAAGCCAG
CD14-rR	CCGCCGTACAATTCCACAT
COL15A1-rF	CCCATTACCCTCGTCTGTGTC
COL15A1-rR	CTGAAGAAGGTCGGTGGGATG
LY96-rF	GAATCTGAGAAGCAACAGTGGT
LY96-rR	CTCAACATGCACAAATCCATTGG
CXCL6-rF	GTTCCATCTCGCCATTCATGC
CXCL6-rR	GCGGCTATGACTGAGGAAGG
CXCL16-rF	CCTTGTCTCTTGCGTTCTTCC
CXCL16-rR	TCCAAAGTACCCTGCGGTATC
SHC4-rF	GAGGAGGTACTTGTTGATGGTG
SHC4-rR	TCTGTAGCTTGAGCCTGGATG
GAPDH-rF	AGACAGCCGCATCTTCTTGT
GAPDH-rR	CTTGCCGTGGGTAGAGTCAT

### Western blotting analysis

The protein levels of toll-like receptor 4 (TLR4), phosphatidylinositol 3-kinase (PI3K), AKT serine (AKT), tumor necrosis factor-α (TNF-α), LYN proto-oncogene, Src family tyrosine kinase (LYN), and caspase-3 were determined by western blotting. The total proteins of ovary tissues were extracted with a protein extraction kit (Beijing Baiolaibo Technology Co. LTD, Beijing, China) and a BCA protein assay kit (Boster Biological Technology Co. LTD, Wuhan, China) was used to determine the concentration of total protein. Furthermore, the proteins were separated by 12% SDS-PAGE, transferred onto a PVDF membrane, and blocked with 5% BSA. The membranes were then incubated with anti-TLR4 (1:2000, Abcam), anti-T-PI3K (1:5000, Abcam), anti-p-PI3K (1:5000, Abcam), anti-T-AKT (1:5000, Abcam), anti-p-Akt (1:5000, Abcam), anti-TNF-α (1:2000, Abcam), anti-IL-1 (1:2000, Abcam), anti-IL-6 (1:2000, Abcam), anti-caspase-3 (1:5000, Abcam), anti-cleaved caspase-3 (1:5000, Abcam), anti-LYN (1:5000, Abcam), and anti-GAPDH (1:5000, Abcam) antibodies, respectively, at room temperature overnight. Thereafter, IgG-HRP was added to the membranes and incubated at room temperature for 2 h. After washing five times, the protein bands were visualized with ECL.

### Statistical analysis

Statistical analyses were performed using GraphPad InStant 3 (San Diego, CA, USA). The data are reported as mean ± standard deviation (SD). One-way analysis of variance was used for comparison among more than two groups, and the Tukey’s test was subsequently performed to assess group differences. For comparisons of two groups, the Student’s *t*-test was applied. A *p*-value of less than 0.05 was considered to indicate a significant difference.

## Results

### Rat ovarian morphology

**Fig. 1:**
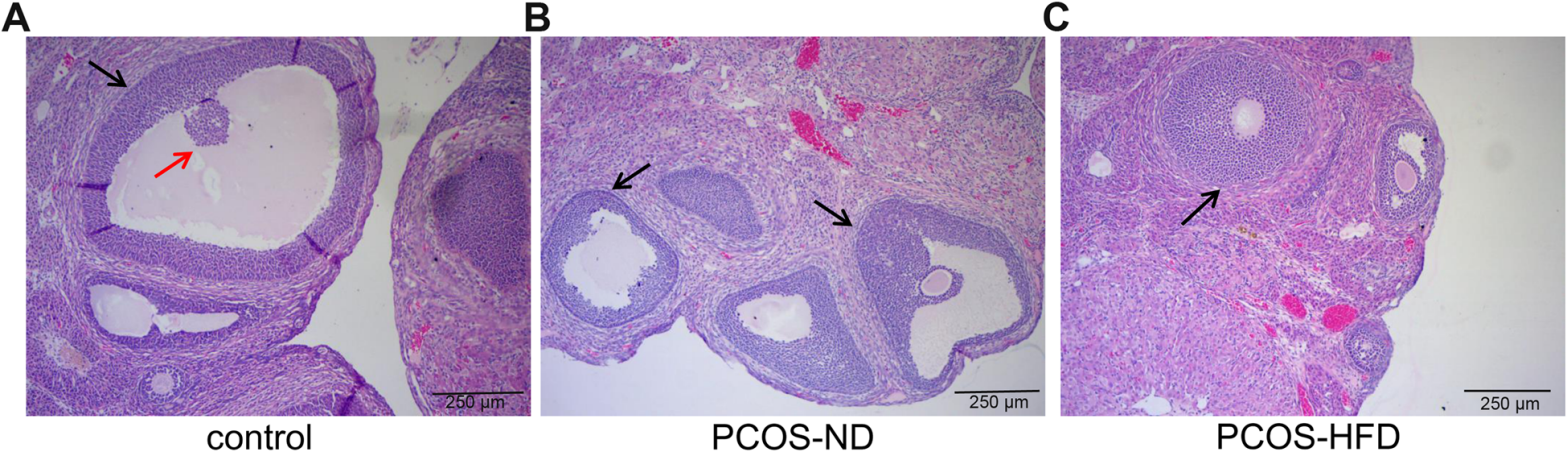
Changes in ovarian morphology in the control, PCOS-ND, and PCOS-HFD group rats. **a** Ovarian morphology of rats in the control group. **b** Ovarian morphology of rats in the PCOS-ND group. **c** Ovarian morphology of rats in the PCOS-HFD group. Black arrow indicates layers of granulosa cells; red arrow indicates oocyte

We compared the rat ovarian morphology in the control, PCOS-ND, and PCOS-HFD groups to identify any changes. HE staining and electron microscopy results showed that the rats in the control group had normal ovarian histology with multiple lutea and eight to nine layers of granulosa cells with the dominant follicle (Fig. 1a, Supplementary Fig. 1). However, in PCOS-ND and PCOS-HFD groups, the ovarian tissues exhibited follicular cysts, a reduction of the granulosa cell layer, and hyperplasia (Fig. 1b, c, Supplementary Fig. 1). These results indicated that the PCOS model was successfully established.

### The effect of BBR on the body weight and HOMA-IR of PCOS rats

**Fig. 2:**
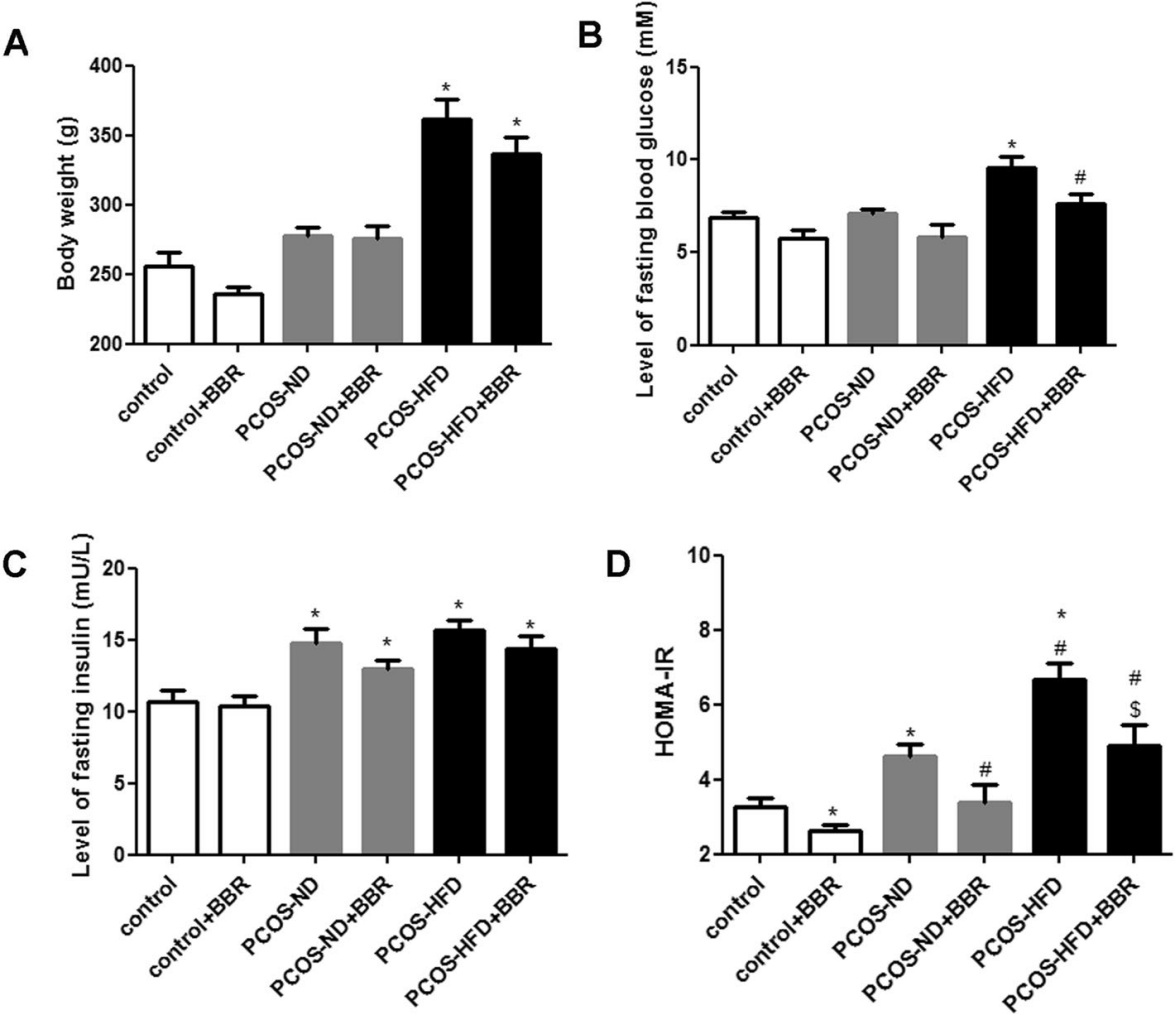
Effects of berberine (BBR) on body weight and homeostasis model assessment of insulin resistance (HOMA-IR) in PCOS rats. **a** Body weight. **b** Level of fasting insulin. **c** Level of fasting blood glucose. **d** HOMA-IR. * *P* < 0.05, compared with the control group. ^#^ *P* < 0.05, compared with the PCOS-ND group. ^$^ *P* < 0.05, compared with the PCOS-HFD group

To evaluate the effects of BBR on PCOS rats, the body weight and HOMA-IR were evaluated. There were no significant differences in the body weight and fasting blood glucose levels among the control, control + BBR, PCOS-ND, and PCOS-ND + BBR groups (*P* > 0.05, Fig. 2a, b). However, the body weights of rats in the PCOS-HFD and PCOS-HFD + BBR groups were significantly higher than those of other groups (*P* < 0.05, Fig. 2a), which indicated that an HFD caused significant weight gain in PCOS rats. Additionally, the fasting blood glucose levels in the PCOS-HFD and PCOS-HFD + BBR groups were 9.57 ± 0.59 and 7.63 ± 0.50 mM, respectively, indicating a significant decrease after BBR treatment in PCOS-HFD rats (*P* < 0.05, Fig. 2b). For fasting insulin, there was no significant difference in its levels between the control and control + BBR groups (*P* > 0.05), while its levels were higher in other groups (*P* < 0.05, Fig. 2c). The intra-assay coefficient of insulin measurements by ELISA was 8.6%. Moreover, compared to the groups not treated with BBR, the HOMA-IR value was significantly decreased after BBR administration (*P* < 0.05, Fig. 2d), indicating that BBR might have a protective effect on IR in PCOS rats.

### The effect of BBR on testosterone in PCOS rats

**Fig. 3:**
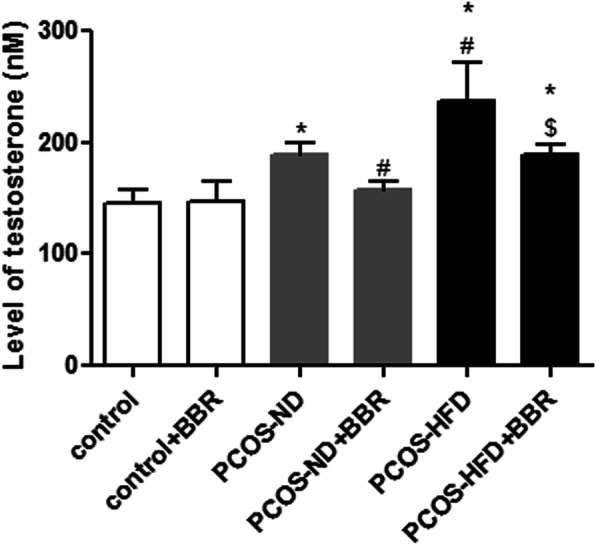
The effect of BBR on testosterone in PCOS rats. * *P* < 0.05, compared with the control group. ^#^ *P* < 0.05, compared with the PCOS-ND group. ^$^ *P* < 0.05, compared with the PCOS-HFD group

The intra-assay coefficient of testosterone determined by ELISA was 7.3%. There was no significant difference in testosterone levels between the control and control + BBR groups (*P* > 0.05, Fig. 3). Additionally, compared with the control group, the testosterone levels in the PCOS rats were significantly greater (*P* < 0.05, Fig. 3). However, after BBR administration, the levels of testosterone decreased (*P* < 0.05); in the PCOS-ND + BBR group, the levels returned to a level which was similar to that of the control group (*P* > 0.05).

### The effect of BBR on cell apoptosis in PCOS rats

**Fig. 4:**
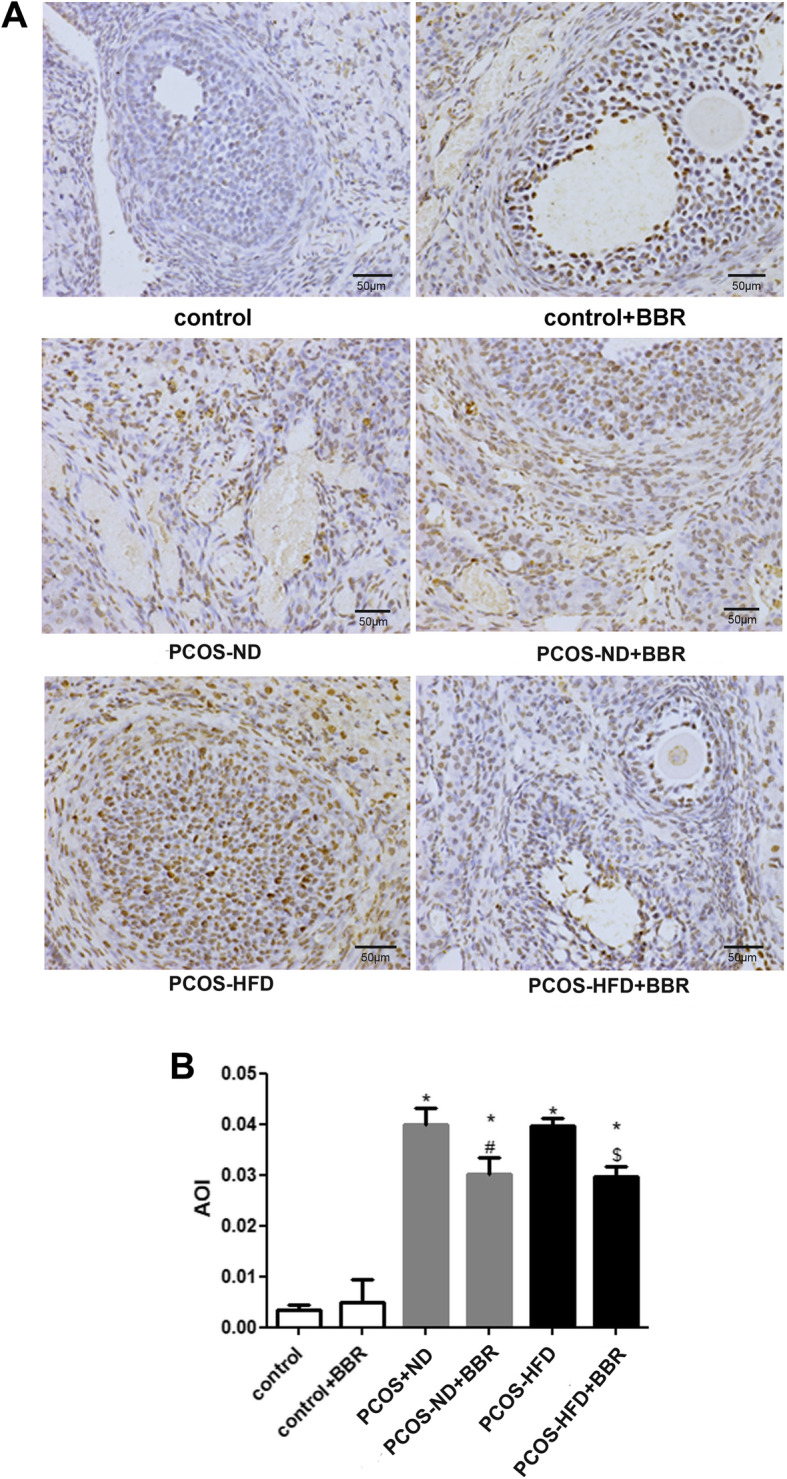
The effects of BBR on cell apoptosis in PCOS rats. **a** Apoptotic cells were observed by using the TUNEL assay kit. The positive apoptotic cell nuclei were dark brown in color, and the non-apoptotic cell nuclei were blue in color. **b** The apoptosis of interest (AOI) was calculated. * *P* < 0.05, compared with the control group. ^#^ *P* < 0.05, compared with the PCOS-ND group. ^$^ *P* < 0.05, compared with the PCOS-HFD group

To investigate the effect of BBR on cell apoptosis in PCOS rats, a TUNEL assay kit was used. The values of apoptosis of interest (AOI) in the control and control + BBR groups were similar (*P* > 0.05), while those in the PCOS-ND and PCOS-HFD groups were significantly higher (*P* < 0.05, Fig. 4). After BBR treatment, the AOI value decreased (*P* < 0.05, Fig. 4), indicating that BBR inhibited cell apoptosis caused by PCOS.

### mRNA expression of *TLR4*, *LYN*, *PI3K*, *Akt*, *NF-kB*, *TNF-α*, *IL-1*, *IL-6*, and *caspase-3* in the ovarian tissues of different groups

**Fig. 5:**
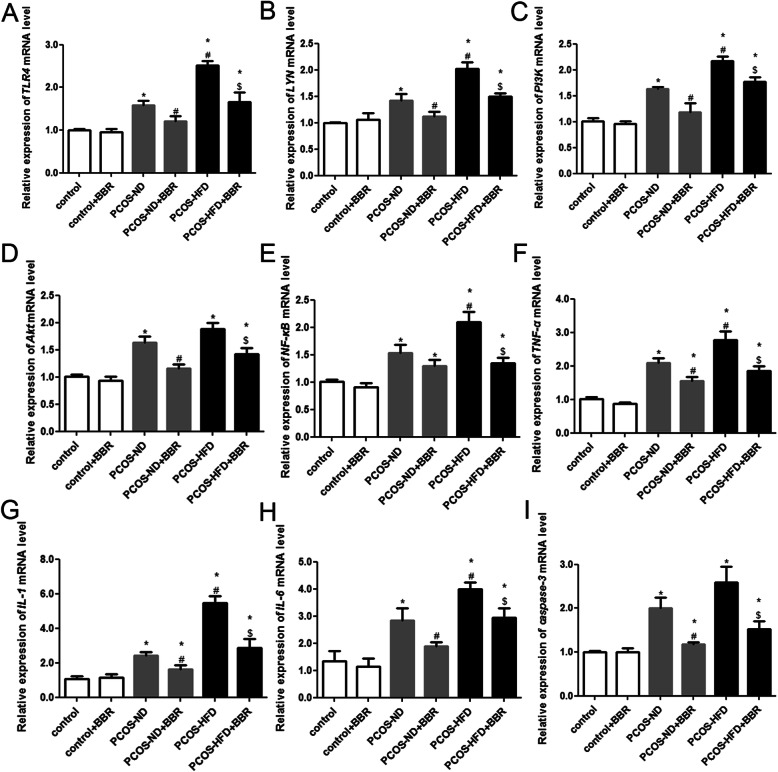
Effects of BBR on mRNA expression of *TLR4*, *LYN*, *PI3K*, *Akt*, *NF-kB*, *TNF-α*, *IL-1*, *IL-6*, and *caspase-3*. The effects of BBR on the mRNA expressions of **a** *TLR4*, **b** *LYN*, **c** *PI3K*, **d** *Akt*, **e** *NF-kB*, **f** *TNF-α*, **g** *IL-1*, **h** *IL-6*, **i** *caspase-3*. * *P* < 0.05, compared with the control group. ^#^ *P* < 0.05, compared with the PCOS-ND group. ^$^ *P* < 0.05, compared with the PCOS-HFD group

RT-qPCR was performed to evaluate the mRNA expression of *TLR4*, *LYN*, *PI3K*, *Akt*, *NF-kB*, *TNF-α*, *IL-1*, *IL-6*, *caspase-3*, *CD14*, *COL15A1*, *LY96*, *CXCL6*, *CXCL16* and *SHC4* among the different groups. The expression levels of *TLR4*, *LYN*, *PI3K*, *Akt*, *NF-kB*, *TNF-α*, *IL-1*, *IL-6*, and *caspase-3* in the control and control + BBR groups were similar (*P* > 0.05, Fig. 5), suggesting that BBR exerted minor side effects on PCOS. However, in PCOS rats (rats in PCOS-ND and PCOS-HFD groups), these levels were significantly higher (*P* < 0.05, Fig. 5); compared with the PCOS-ND group, the expression levels of *TLR4*, *LYN*, *PI3K*, *NF-kB*, *TNF-α*, *IL-1*, and *IL-6* in the PCOS-HFD group were significantly higher (*P* < 0.05, Fig. 5). Further, after BBR administration, the expression levels significantly decreased (*P* < 0.05, Fig. 5), and this decrease was greater in the PCOS-HFD group compared to that in the PCOS-ND group, indicating that BBR demonstrated a better therapeutic effect on PCOS-HFD rats. For *CD14*, *COL15A1*, *LY96*, *CXCL6*, *CXCL16* and *SHC4*, there were no significant differences in their expression among these different groups (*P* > 0.05, Supplementary Fig. 2).

### Protein levels of TLR4, LYN, PI3K, p-PI3K, Akt, p-Akt, TNF-α, IL-1, IL-6, caspase-3, and cleaved caspase-3 in the ovarian tissues of different groups

**Fig. 6:**
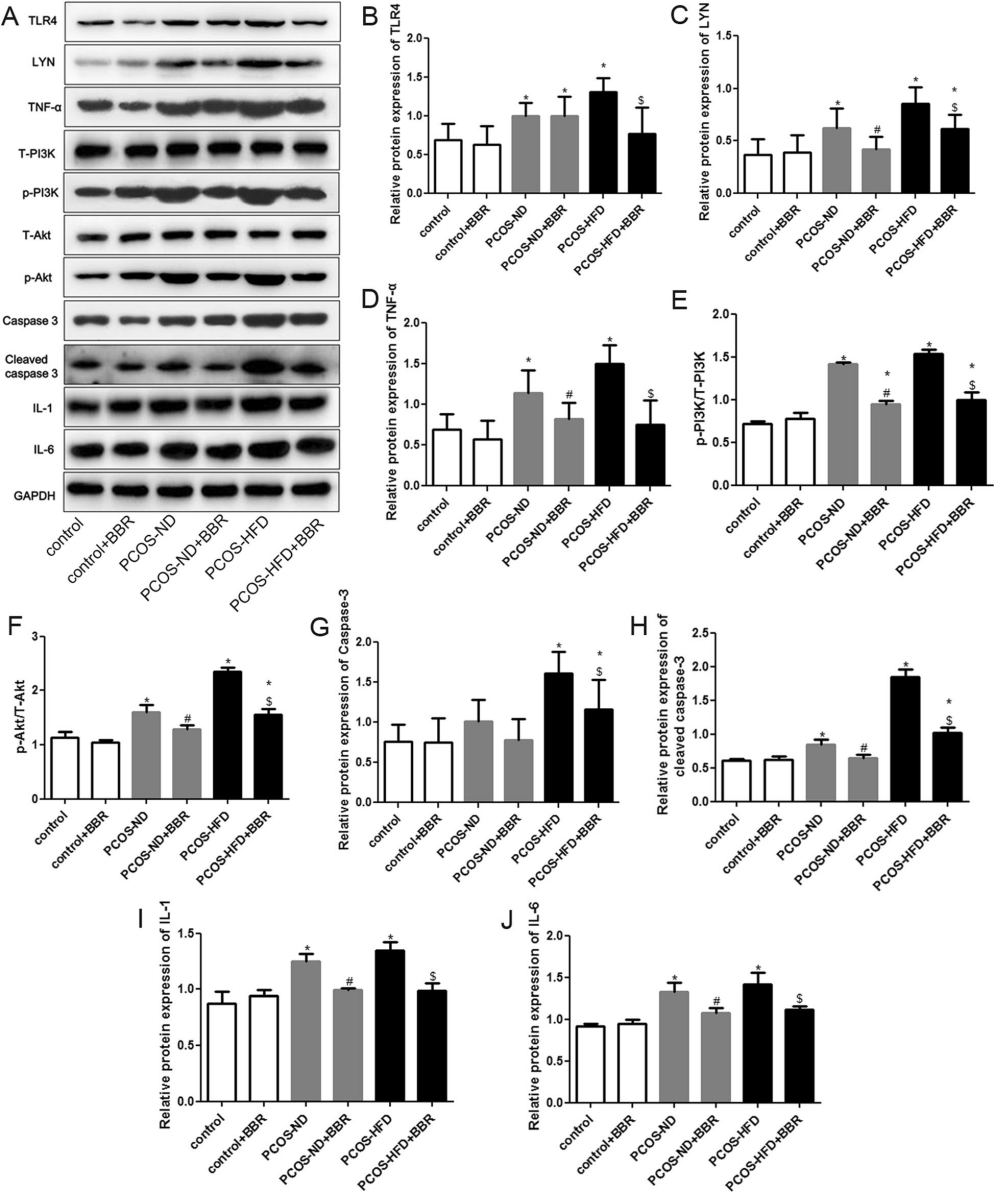
Effects of BBR on protein expression of TLR4, LYN, TNF-α, p-PI3K, T-PI3K, p-Akt, T-Akt, caspase-3, cleaved caspase-3, IL-1, and IL-6. **a** Effects of BBR on the protein expressions of related genes determined by western blotting. **b** Protein levels of TRL4. **c** Protein levels of LYN. **d** Protein levels of TNF-α. **e** Levels of p-PI3K/T-PI3K in different groups. **f** Levels of p-Akt/T-Akt in different groups. **g** Protein levels of caspase-3. **h** Protein levels of cleaved caspase-3. **i** Protein levels of IL-1. **j** Protein levels of IL-6. * *P* < 0.05, compared with the control group. ^#^ *P* < 0.05, compared with the PCOS-ND group. ^$^ *P* < 0.05, compared with the PCOS-HFD group

Western blotting results showed that there were no significant differences in the protein levels of related genes in ovarian tissues (Fig. 6). The protein levels of TLR4 in PCOS rats were higher than those in control rats. Meanwhile, these levels in the PCOS-ND + BBR group were similar to those of the PCOS-ND group (*P* > 0.05). Finally, the protein levels in the PCOS-HFD + BBR group were lower compared to those in the PCOS-HFD group (*P* < 0.05, Fig. 6a, b). Furthermore, the protein expressions of LYN, TNF-α, IL-1, and IL-6 in PCOS rats were significantly upregulated compared to those of the control rats (*P* < 0.05). Additionally, after BBR treatment, the expression levels were significantly downregulated (*P* < 0.05, Fig. 6a, c, d, i, j). The levels of p-PI3K/T-PI3K and p-Akt/T-Akt in the PCOS rats were significantly higher than those in the control group (*P* < 0.05), whereas these levels were evidently decreased after BBR treatment (*P* < 0.05, Fig. 6a, e, f). Additionally, there were no significant differences in caspase-3 protein levels among the control, PCOS-ND, and PCOS-ND + BBR groups (*P* > 0.05). However, compared with the control group, the expression of caspase-3 in the PCOS-HFD group was significantly upregulated (*P* < 0.05), while after BBR administration, its expression was downregulated in the PCOS-HFD + BBR group (*P* < 0.05, Fig. 6a, g). For cleaved caspase-3, its protein levels significantly increased in the PCOS rats compared to those of the control rats. Meanwhile, after BBR treatment, the protein levels were markedly decreased (*P* < 0.05, Fig. 6a, h).

## Discussion

PCOS is a complex disease with multiple factors and various clinical manifestations that seriously affects women’s health. BBR, a traditional Chinese herbal medicine, is used as an insulin sensitizer to improve glucose and lipid metabolism [22]. In this study, a PCOS with IR model was successfully established by injecting rats with DHEA and by feeding an HFD. We found that BBR reduced the weight gain caused by the HFD, thereby inhibiting the levels of IR and testosterone. Additionally, RT-qPCR and western blotting results showed that BBR alleviated PCOS by suppressing cell apoptosis and by regulating the expression levels of related genes, including *TLR4*, *LYN*, *PI3K*, *Akt*, *NF-kB*, *TNF-α*, *IL-1*, *IL-6*, and *caspase-3*.

IR, a major characteristic of PCOS, has been observed in 20–40% of the women with PCOS [23]. Hyperandrogenemia, another central component of PCOS, is evaluated by measuring the levels of total testosterone in PCOS rats [24]. A study by Chen et al. found that Quyu Huatan decoction not only ameliorated the secretion of testosterone but also regulated the body’s metabolism of glucose and lipids in patients with PCOS [25]. In this study, BBR reduced HOMA-IR in PCOS rats and might have improved the symptoms of PCOS by inhibiting the production of testosterone, which indicated that BBR might be worth clinical promotion and application.

Additionally, an increasing number of studies continue to demonstrate that PCOS is associated with the inflammatory response and cell apoptosis [26, 27]. Therefore, RT-qPCR and western blotting were used in this study to explore the expression of inflammation-related genes. We showed that BBR regulated the expression levels of *TLR4*, *LYN*, *NF-kB*, *TNF-α*, *IL-1*, and *IL-6* caused by PCOS. *TLR4* plays an important role in proinflammatory signaling and its levels are significantly higher in rats with PCOS compared to those without PCOS [28]. Further, *TLR4* binds to its receptor and activates *NF-kB* to produce proinflammatory cytokines, including *TNF-α*, *IL-1*, and *IL-6* [11, 29]. In this study, TNF-α, IL-1, and IL-6 were upregulated in the PCOS rats. Moreover, after BBR treatment, these levels were significantly downregulated. These results indicated that BBR reduced the inflammatory response of PCOS rats. Additionally, *LYN*, encoding tyrosine-protein Lyn, is essential for the generation of the inflammatory environment and for the activation of the innate immune response mediated by *NF-kB* [30, 31]. Comparing the results of the present study with those of previous studies, it is apparent that BBR may alleviate the inflammatory response of PCOS rats by regulating the expressions of *TLR4*, *LYN*, *NF-kB*, *TNF-α*, *IL-1*, and *IL-6*, thus improving the symptoms of PCOS.

The PI3K/Akt signaling pathway changes with resistance to insulin as well as androgen and follicular development [32]. Qiu et al. indicated that Liuwei Dihuang pills improved IR by acting on the PI3K/Akt signaling pathway, thus alleviating the symptoms of PCOS [33]. Additionally, the upregulation of WNT5a may enhance the inflammatory response and oxidative stress in PCOS via the PI3K/Akt/NF-kB signaling pathway. In the present study, we found that BBR mediated the levels of p-PI3K/T-PI3K, p-Akt/T-Akt, and NF-kB; thus, we suggest that BBR may improve PCOS via the PI3K/Akt/NF-kB signaling pathway. However, the specific mechanism is currently unclear and requires further investigation.

Cell apoptosis in ovarian tissues plays an important role in PCOS [34–36]. Therefore, TUNEL, RT-qPCR, and western blotting were used to investigate cell apoptosis and the expression of *caspase-3* and *cleaved caspase-3*. *Caspase-3*, the main executor of apoptosis, is essential for chromatin condensation, DNA fragmentation, and nuclear and plasma membrane vacuolization, and is also associated with different processes of apoptotic body formation [34]. A study by Salehi et al. showed that *caspase-3* was overexpressed in patients with PCOS, and its higher expression might result in higher apoptosis levels [34]. Elevated levels of cleaved caspase-3 are closely associated with cell apoptosis [37]. In the present study, the expression of caspase-3 and cleaved caspase-3 was higher in PCOS rats than that in control rats, while BBR downregulated the expression of *caspase-3* and *cleaved caspase-3* as well as inhibited ovarian cell apoptosis.

However, there are some limitations to this study. The changes in steroidogenic enzymes in serum require further investigation, and the influence of BBR on estrous cycle and fertility in DHEA-treated rats also requires confirmation. Additionally, further studies are needed to elucidate the apoptosis of different cells (granulosa cells, cumulus cells, and stroma) in the ovaries.

In conclusion, BBR reduces IR and testosterone levels in PCOS rats and exerts a protective effect on PCOS. The underlying mechanism of BBR action may be involved in the regulation of the expression of *TLR4*, *LYN*, *PI3K*, *Akt*, *NF-kB*, *TNF-α*, *IL-1*, *IL-6*, and *caspase-3*, thus inhibiting the inflammatory response as well as cell apoptosis. Our findings provide a novel effective therapeutic strategy for PCOS.

## Supplementary Information

**Additional file 1: Supplementary Fig. 1** Ovarian morphology of rats in the control group, PCOS-ND group, and PCOS-HFD group.

**Additional file 2: Supplementary Fig. 2** The mRNA expressions of *CD14*, *COL15A1*, *LY96*, *CXCL6*, *CXCL16* and *SHC4* in different groups.

## Data Availability

All data generated or analyzed during this study are included in this published article.

## Abbreviations

PCOS: Polycystic ovary syndrome
BBR: Berberine
IR: Insulin resistance
DHEA: Dehydroepiandrosterone
SLAC: Shanghai Laboratory Animal Centre
FPG: Fasting plasma glucose
FINS: Fasting insulin
HOMA-IR: Homeostasis model assessment of insulin resistance
HE: Hematoxylin and eosin
TLR4: Toll-like receptor 4
PI3K: Phosphatidylinositol 3-kinase
TNF-α: Tumor necrosis factor-α
LYN: Src family tyrosine kinase
SD: Standard deviation

## References

[1] Chen Y, Yang T, Hao C, Zhao J (2018). A retrospective study of Letrozole treatment prior to human chorionic gonadotropin in women with polycystic ovary syndrome undergoing in vitro fertilization at risk of ovarian Hyperstimulation syndrome. Med Sci Monitor.

[2] Hamilton KP, Zelig R, Parker AR, Haggag A (2019). Insulin resistance and serum magnesium concentrations among women with polycystic ovary syndrome. Curr Dev Nutr.

[3] Wang J, Wu D, Guo H, Li M (2019). Hyperandrogenemia and insulin resistance: the chief culprit of polycystic ovary syndrome. Life Sci.

[4] Harris HR, Terry KL (2016). Polycystic ovary syndrome and risk of endometrial, ovarian, and breast cancer: a systematic review. Fertil Res Pract.

[5] Kakoly NS, Earnest A, Teede HJ, Moran LJ, Joham AE (2019). The impact of obesity on the incidence of type 2 diabetes among women with polycystic ovary syndrome. Diabetes Care.

[6] Karthik S, Vipin VP, Kapoor A, Tripathi A, Shukla M, Dabadghao P (2019). Cardiovascular disease risk in the siblings of women with polycystic ovary syndrome. Hum Reprod.

[7] Macut D, Mladenovic V, Bjekic-Macut J, Livadas S, Stanojlovic O, Hrncic D, Rasic-Markovic A, Milutinovic DV, Andric Z (2020). Hypertension in polycystic ovary syndrome: novel insights. Curr Hypertens Rev.

[8] Cunningham P (2017). Pathophysiology, diagnosis and treatment of polycystic ovary syndrome. Nurs Stand.

[9] Ramezani Tehrani F, Amiri M (2019). Polycystic ovary syndrome in adolescents: challenges in diagnosis and treatment. Int J Endocrinol Metab.

[10] Hong Y, Yin Y, Tan Y, Hong K, Zhou H (2019). The Flavanone, Naringenin, modifies antioxidant and Steroidogenic enzyme activity in a rat model of Letrozole-induced polycystic ovary syndrome. Med Sci Monitor.

[11] Wang Z, Zhai D, Zhang D, Bai L, Yao R, Yu J, Cheng W, Yu C (2017). Quercetin decreases insulin resistance in a polycystic ovary syndrome rat model by improving inflammatory microenvironment. Reprod Sci.

[12] Xiao D, Liu Z, Zhang S, Zhou M, He F, Zou M, Peng J, Xie X, Liu Y, Peng D (2018). Berberine derivatives with different pharmacological activities via structural modifications. Mini Rev Medicinal Chem.

[13] Imanshahidi M, Hosseinzadeh H (2008). Pharmacological and therapeutic effects of Berberis vulgaris and its active constituent, berberine. Phytotherapy Res.

[14] Wang Y, Fu X, Xu J, Wang Q, Kuang H (2016). Systems pharmacology to investigate the interaction of berberine and other drugs in treating polycystic ovary syndrome. Sci Rep.

[15] Zhao L, Li W, Han F, Hou L, Baillargeon JP, Kuang H, Wang Y, Wu X (2011). Berberine reduces insulin resistance induced by dexamethasone in theca cells in vitro. Fertil Steril.

[16] Zhang X, Zhao Y, Zhang M, Pang X, Xu J, Kang C, Li M, Zhang C, Zhang Z, Zhang Y, Li X, Ning G, Zhao L (2012). Structural changes of gut microbiota during berberine-mediated prevention of obesity and insulin resistance in high-fat diet-fed rats. PLoS One.

[17] Wei W, Zhao H, Wang A, Sui M, Liang K, Deng H, Ma Y, Zhang Y, Zhang H, Guan Y (2012). A clinical study on the short-term effect of berberine in comparison to metformin on the metabolic characteristics of women with polycystic ovary syndrome. Eur J Endocrinol.

[18] Abramovich D, Irusta G, Bas D, Cataldi NI, Parborell F, Tesone M (2012). Angiopoietins/TIE2 system and VEGF are involved in ovarian function in a DHEA rat model of polycystic ovary syndrome. Endocrinology.

[19] Yu J, Zhai D, Hao L, Zhang D, Bai L, Cai Z, Yu C (2014). Cryptotanshinone reverses reproductive and metabolic disturbances in PCOS model rats via regulating the expression of CYP17 and AR. Evid Based Complement Alternat Med.

[20] Bowe JE, Franklin ZJ, Hauge-Evans AC, King AJ, Persaud SJ, Jones PM (2014). Metabolic phenotyping guidelines: assessing glucose homeostasis in rodent models. J Endocrinol.

[21] Coniglio RI, Merono T, Montiel H, Malaspina MM, Salgueiro AM, Otero JC, Ferraris R, Schreier L, Brites F, Gomez Rosso L (2012). HOMA-IR and non-HDL-C as predictors of high cholesteryl ester transfer protein activity in patients at risk for type 2 diabetes. Clin Biochem.

[22] Li MF, Zhou XM, Li XL (2018). The effect of Berberine on polycystic ovary syndrome patients with insulin resistance (PCOS-IR): a meta-analysis and systematic review. Evid Based Complement Alternat Med.

[23] Bhathena RK (2011). Insulin resistance and the long-term consequences of polycystic ovary syndrome. J Obstet Gynaecol.

[24] Karakas SE (2017). New biomarkers for diagnosis and management of polycystic ovary syndrome. Clin Chim Acta.

[25] Chen WJ, Wang FF (2016). Effect of Quyu Huatan decoction on lipid metabolism and hormone levels of patients with polycystic ovary syndrome. Zhongguo Zhong Yao Za Zhi.

[26] Shorakae S, Teede H, de Courten B, Lambert G, Boyle J, Moran LJ (2015). The emerging role of chronic low-grade inflammation in the pathophysiology of polycystic ovary syndrome. Semin Reprod Med.

[27] Tao T, Wu P, Wang Y, Liu W (2018). Comparison of glycemic control and beta-cell function in new onset T2DM patients with PCOS of metformin and saxagliptin monotherapy or combination treatment. BMC Endocr Disord.

[28] Wang Y, He J, Yang J (2018). Eicosapentaenoic acid improves polycystic ovary syndrome in rats via sterol regulatory element-binding protein 1 (SREBP-1)/toll-like receptor 4 (TLR4) pathway. Med Sci Monitor.

[29] Bhaskar S, Shalini V, Helen A (2011). Quercetin regulates oxidized LDL induced inflammatory changes in human PBMCs by modulating the TLR-NF-kappaB signaling pathway. Immunobiology.

[30] Kovacs M, Nemeth T, Jakus Z, Sitaru C, Simon E, Futosi K, Botz B, Helyes Z, Lowell CA, Mocsai A (2014). The Src family kinases Hck, Fgr, and Lyn are critical for the generation of the in vivo inflammatory environment without a direct role in leukocyte recruitment. J Exp Med.

[31] Toubiana J, Rossi AL, Belaidouni N, Grimaldi D, Pene F, Chafey P, Comba B, Camoin L, Bismuth G, Claessens YE, Mira JP, Chiche JD (2015). Src-family-tyrosine kinase Lyn is critical for TLR2-mediated NF-kappaB activation through the PI 3-kinase signaling pathway. Innate Immunity.

[32] Li T, Mo H, Chen W, Li L, Xiao Y, Zhang J, Li X, Lu Y (2017). Role of the PI3K-Akt signaling pathway in the pathogenesis of polycystic ovary syndrome. Reprod Sci.

[33] Qiu Z, Dong J, Xue C, Li X, Liu K, Liu B, Cheng J, Huang F (2020). Liuwei Dihuang pills alleviate the polycystic ovary syndrome with improved insulin sensitivity through PI3K/Akt signaling pathway. J Ethnopharmacol.

[34] Salehi E, Aflatoonian R, Moeini A, Yamini N, Asadi E, Khosravizadeh Z, Tarzjani MD, Harat ZN, Abolhassani F (2017). Apoptotic biomarkers in cumulus cells in relation to embryo quality in polycystic ovary syndrome. Arch Gynecol Obstet.

[35] Uyanikoglu H, Sabuncu T, Dursun H, Sezen H, Aksoy N (2017). Circulating levels of apoptotic markers and oxidative stress parameters in women with polycystic ovary syndrome: a case-controlled descriptive study. Biomarkers.

[36] Yang R, Chen J, Wang L, Deng A (2019). LncRNA BANCR participates in polycystic ovary syndrome by promoting cell apoptosis. Mol Med Rep.

[37] Zheng L, Wang W, Ni J, Mao X, Song D, Liu T, Wei J, Zhou H (2017). Role of autophagy in tumor necrosis factor-alpha-induced apoptosis of osteoblast cells. J Investig Med.

