# Supplementary figures and images for: Berberine exerts a protective effect on rats with polycystic ovary syndrome by inhibiting the inflammatory response and cell apoptosis
Source: Reprod Biol Endocrinol. 2021 Jan 7;19:3. doi: 10.1186/s12958-020-00684-y (PMC7789273; doi:10.1186/s12958-020-00684-y)

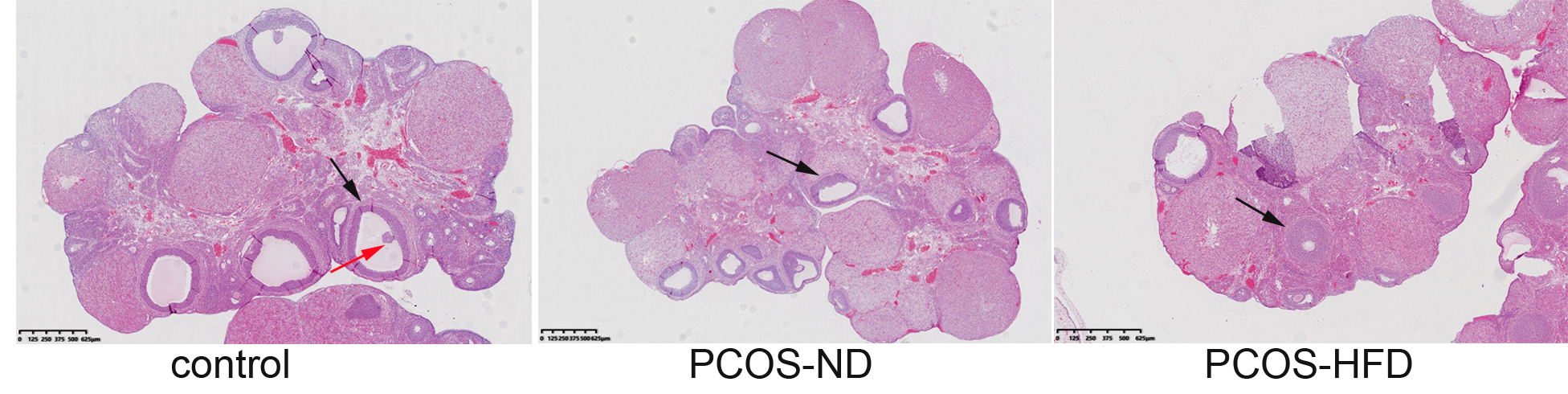

Supplement: Supplementary file 1 — Additional file 1: Supplementary Fig. 1 Ovarian morphology of rats in the control group, PCOS-ND group, and PCOS-HFD group. [file 12958_2020_684_MOESM1_ESM.tif]

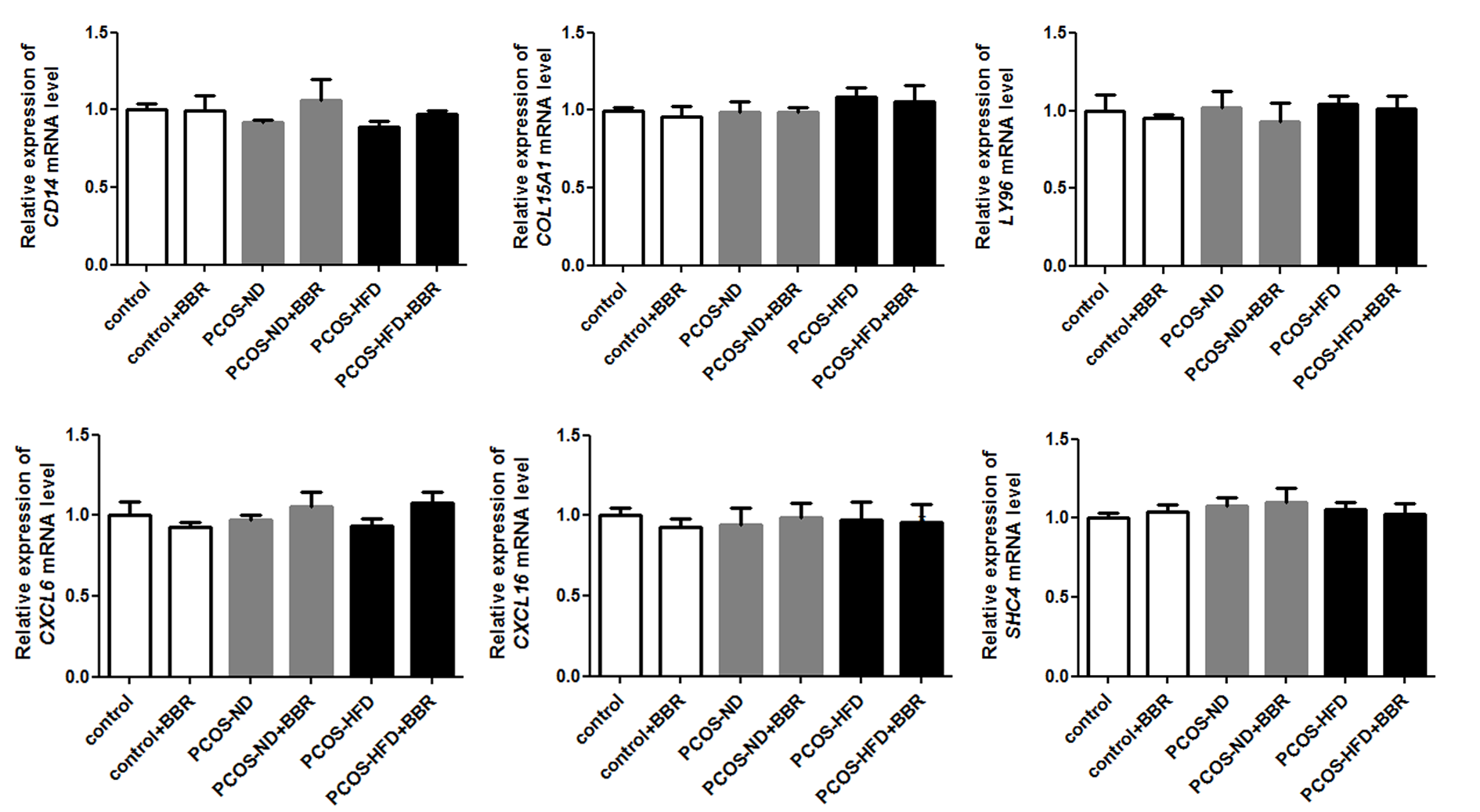

Supplement: Supplementary file 2 — Additional file 2: Supplementary Fig. 2 The mRNA expressions of CD14, COL15A1, LY96, CXCL6, CXCL16 and SHC4 in different groups. [file 12958_2020_684_MOESM2_ESM.tif]
